# Hydrogen Gas Attenuates Hypoxic-Ischemic Brain Injury via Regulation of the MAPK/HO-1/PGC-1a Pathway in Neonatal Rats

**DOI:** 10.1155/2020/6978784

**Published:** 2020-02-13

**Authors:** Peipei Wang, Mingyi Zhao, Zhiheng Chen, Guojiao Wu, Masayuki Fujino, Chen Zhang, Wenjuan Zhou, Mengwen Zhao, Shin-ichi Hirano, Xiao-Kang Li, Lingling Zhao

**Affiliations:** ^1^Department of Pediatrics, the Third Xiangya Hospital, Central South University, Changsha, China; ^2^Division of Transplantation Immunology, National Research Institute for Child Health and Development, Tokyo, Japan; ^3^AIDS Research Center, National Institute of Infectious Diseases, Tokyo, Japan; ^4^MiZ Co., Ltd., Kanagawa, Japan

## Abstract

Neonatal hypoxic-ischemic encephalopathy (HIE) is a leading cause of death in neonates with no effective treatments. Recent advancements in hydrogen (H_2_) gas offer a promising therapeutic approach for ischemia reperfusion injury; however, the impact of this approach for HIE remains a subject of debate. We assessed the therapeutic effects of H_2_ gas on HIE and the underlying molecular mechanisms in a rat model of neonatal hypoxic-ischemic brain injury (HIBI). H_2_ inhalation significantly attenuated neuronal injury and effectively improved early neurological outcomes in neonatal HIBI rats as well as learning and memory in adults. This protective effect was associated with initiation time and duration of sustained H_2_ inhalation. Furthermore, H_2_ inhalation reduced the expression of Bcl-2-associated X protein (BAX) and caspase-3 while promoting the expression of Bcl-2, nuclear factor erythroid-2-related factor 2, and heme oxygenase-1 (HO-1). H_2_ activated extracellular signal-regulated kinase and c-Jun N-terminal protein kinase and dephosphorylated p38 mitogen-activated protein kinase (MAPK) in oxygen-glucose deprivation/reperfusion (OGD/R) nerve growth factor-differentiated PC12 cells. Inhibitors of MAPKs blocked H_2_-induced HO-1 expression. HO-1 small interfering RNA decreased the expression of peroxisome proliferator-activated receptor gamma coactivator 1-alpha (PGC-1*α*) and sirtuin 1 (SIRT1) and reversed the protectivity of H_2_ against OGD/R-induced cell death. These findings suggest that H_2_ augments cellular antioxidant defense capacity through activation of MAPK signaling pathways, leading to HO-1 expression and subsequent upregulation of PGC-1*α* and SIRT-1 expression. Thus, upregulation protects NGF-differentiated PC12 cells from OGD/R-induced oxidative cytotoxicity. In conclusion, H_2_ inhalation exerted protective effects on neonatal rats with HIBI. Early initiation and prolonged H_2_ inhalation had better protective effects on HIBI. These effects of H_2_ may be related to antioxidant, antiapoptotic, and anti-inflammatory responses. HO-1 plays an important role in H_2_-mediated protection through the MAPK/HO-1/PGC-1*α* pathway. Our results support further assessment of H_2_ as a potential therapeutic for neurological conditions in which oxidative stress and apoptosis are implicated.

## 1. Background

Neonatal hypoxic-ischemic encephalopathy (HIE) is a severe disease with high neonatal morbidity and mortality; 25% of HIE survivors have permanent neurological defects [1]. Before irreversible brain injury occurs, the specific pathological process of hypoxia-ischemia (HI) occurs through a combination of multiple mechanisms [2, 3]. The severity and duration of these mechanisms determine the extent of brain injury in HIE [4].

Molecular hydrogen (H_2_) easily penetrates the blood-brain barrier and is a novel antioxidant [5]. Previous studies have shown that H_2_ mitigates ischemia/reperfusion- (I/R-) induced injury to different organs [6–8]. However, the neuroprotective effects of H_2_ treatment on hypoxic-ischemic brain injury (HIBI) are controversial. For example, a recent study showed that H_2_-enriched water exerts neuroprotective effects on brain tissue after induction of HIBI [9]. However, another study found no protective effects [10].

Oxidative stress is a well-recognized consequence of HIE and is considered an important contributor to early brain injury after HI [11] working in combination with inflammation [12]. Growing evidence suggests that oxidative stress and neuroinflammation underpin a diverse range of central nervous system (CNS) diseases including stroke, traumatic brain injury, multiple sclerosis, Alzheimer's, Parkinson's, and other neurodegenerative diseases [13–15]. Heme oxygenase-1 (HO-1) is an important component of the cellular defense enzyme that is induced by and acts against oxidant-induced I/R injury [16]. HO-1 overexpression may also affect the regulation of apoptotic pathway genes such as B-cell lymphoma 2 (Bcl-2), Bcl-2-associated X protein (BAS), and caspases [17]. HO-1 has antineuroinflammatory and neuroprotective properties in the CNS [18, 19]. Thus, therapies targeting HO-1 may be potential treatments for protection against inflammation, oxidative stress, and apoptosis after HI.

Multiple signaling kinases related to cell survival and proliferation reportedly regulate the nuclear translocation of HO-1. Mitogen-activated protein kinases (MAPKs) are some of the most common signaling pathways, which serve to coordinate the cellular response to a variety of extracellular stimuli [20]. These are well characterized in mammals and include c-Jun N-terminal kinase (JNK), p38 MAPK, and extracellular signal-regulated kinase (ERK) [21, 22]. Activation of MAPKs modulates HO-1 expression [23].

In this study, we evaluated the neuroprotective effects of H_2_ on neonatal HIBI rats through behavioral tests, immunofluorescence, and western blot analysis to determine the appropriate therapeutic window for of H_2_. *In vivo*, we also tested the expression of high-mobility group protein B1 (HMGB1) and toll-like receptor 4 (TLR-4), the apoptosis-related factors Bcl-2/BAX-caspase3, and the oxidative stress signaling molecules p38 MAPK/Nrf2/HO-1. Then, we investigated the potential MAPK/HO-1/peroxisome proliferator-activated receptor gamma coactivator 1-alpha (PGC-1*α*) signaling pathway in nerve growth factor- (NGF-) differentiated PC12 cells after oxygen-glucose deprivation/reperfusion (OGD/R).

## 2. Methods

### 2.1. Experimental Design In Vivo

**Figure 1:**
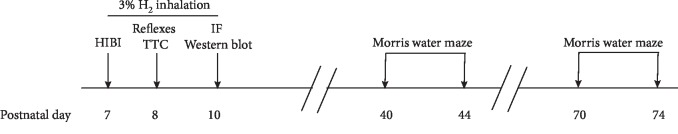
Experimental diagram *in vivo.*

A total of 320 neonatal rats were randomly divided into the following eight groups (*n* = 40/group): HIBI, HIBI+H_2_-30 min, HIBI+H_2_-60 min, HIBI+H_2_-90 min, HIBI+H_2_-90 min (12 h), HIBI+H_2_-90 min (24 h), sham surgery, and control. Animals in the HIBI and all HIBI+H_2_ treatment groups were rats with induced HIBI. After establishing the HIBI model, neonatal rats in the HIBI+H_2_-30 min, HIBI+H_2_-60 min, and HIBI+H_2_-90 min groups were immediately placed in a box for 30, 60, or 90 min of H_2_ inhalation, respectively. This was followed by twice daily H_2_ inhalation (30, 60, or 90 min each time) over the next 3 days (7 : 00–9 : 00 am and 7 : 00–9 : 00 pm). Animals in the HIBI+H_2_-90 min (12 h) and HIBI+H_2_-90 min (24 h) groups underwent 3% H_2_ inhalation for 90 min at 12 and 24 h, respectively, after induction of HI. This was followed by twice daily H_2_ inhalation for 90 min for 3 days. Animals in the sham surgery group underwent left carotid artery isolation without ligation and hypoxia induction. Animals in the control group were healthy neonatal rats without induction of HIBI. Detection of early neurological reflexes was performed ~24 h after HIBI induction, and then some neonatal rats were subjected to triphenyltetrazolium chloride (TTC) staining (*n* = 8/group). At 96 h after inducing HIBI, brain samples from neonatal rats were collected for western blotting, paraffin-embedded, and sectioned for terminal deoxynucleotidyl transferase dUTP nick end labeling (TUNEL) and immunofluorescence staining. In addition, some of the neonatal rats (*n* = 8/group) in adulthood (postnatal days 70–74) underwent testing in a Morris water maze (Figure 1). The study was performed in a blinded and randomized matter with respect to treatment administration and histological and functional assessments.

### 2.2. Experimental Animals

Neonatal Sprague-Dawley rats (males and females, 7 days old, 12–18 g), were provided by the Department of Laboratory Animals, Central South University (Hunan Province, China). All pups were housed with their mothers before and after the experiments in a barrier environment (all measurement indicators were in conformity with the national standard “Experimental Animal Environment and Facilities GB14925, 2010”). All of the experiments were conducted in accordance with the *Guide for the Care and Use of Laboratory Animals* (National Institute of Health Publication No. 80-23, revised 1996), and approved by the Center for Medical Ethics of the Third Xiangya Hospital (Hunan, China). Sincere efforts were made to minimize animal suffering and to reduce the number of animals used.

### 2.3. HIBI Modeling and H_2_ Inhalation

Neonatal rats at 7 days of age were anesthetized with isoflurane inhalation. This was followed by making a 0.7 cm incision to the left of the cervical midline to separate the left common carotid artery, which was severed after bilateral ligation. Surgical suture 6-0 was used to close the incision. The entire surgical procedure was completed within 8 min. Neonatal rats recovered in the same cage as their mother for 2 h after surgery. Next, they were placed in an anoxic tank (Gaoke Gas Technology Co. Ltd., Changsha, China) containing 8% oxygen and 92% nitrogen at 37°C to induce hypoxia for 100 min. After hypoxic exposure, neonatal rats were allowed to recover or underwent H_2_ inhalation. The inhaled gas for H_2_ intervention was a mixture of H_2_ and air. H_2_ was generated by electrolysis of water in the MHG-2000 Hydrogen Generator (MiZ Co. Ltd., Kanagawa, Japan) at a concentration of 3%.

### 2.4. TTC Staining

The infarct volume was determined by TTC staining. Using this method, the brain sections were prepared as follows. First, the brains were removed and frozen at -20°C for 10 min. Next, consecutive 2 mm coronal sections were obtained by slicing the brains with a Brain Matrix (ASI Instruments, Warren, MI, USA). The subsequent incubation of the sections was performed in a dark environment with a 30 min immersion in 2% TTC solution at 37°C. TTC stained normal areas of brain deep red but did not stain the infarcted tissue. Infarction volumes were measured and analyzed with ImageJ software (version 1.61; NIH Image, Bethesda, MD, USA).

### 2.5. Immunofluorescence

After the last H_2_ treatment, rats were perfused with cold 0.1 M phosphate-buffered saline (PBS, pH 7.4) through the left ventricle. The brains were fixed in 4% paraformaldehyde overnight. Fixed brain tissues were further processed by dehydration in graded ethanol prior to paraffin embedding. Finally, the blocks were serially cut into 5 *μ*m thick sections and stored at 4°C. After deparaffinization, the sections were pretreated within antigen unmasking solution (Vector Laboratories, Burlingame, CA, USA) for 15 min in the microwave oven (medium fire for 8 min, ceased fire for 8 min, and low fire for 7 min). After washing with PBS, the sections were quenched with spontaneous fluorescence quenching agent (Servicebio, Wuhan, China) for 5 min at room temperature. Nonspecific binding sites were blocked by incubating the sections in 5% bovine serum albumin for 30 min. This was followed by an overnight incubation at 4°C with the following primary antibodies: anti-glial fibrillary acidic protein (GFAP) (1 : 1000; Sigma, St. Louis, MO, USA), anti-ionized calcium-binding adaptor molecule 1 (1 : 1000; Wako, Osaka, Japan), anti-HMGB1 (1 : 500; Abcam, Cambridge, MA, USA), anti-TLR4 (1 : 100; Servicebio), anti-Nrf2 (1 : 100; Abcam), anti-HO-1 (1 : 100; Abcam), anti-BAX (1 : 100; Abcam), anti-Bcl2 (1 : 100; Abcam), anti-caspase3 (1 : 100; Servicebio), and anti-p-p38 MAPK (1 : 100, Abcam). Then the sections were immersed in PBS and incubated with the secondary antibody (Servicebio) for 50 min at room temperature. Finally, the sections were incubated for 10 min with DAPI. After washing with PBS, the sections were sealed with antifluorescence quenching sealant and scanned using Pannoramic MIDI (3DHISTECH Ltd., Budapest, Hungary). CaseViewer software was used to merge the stained images and integrate the fluorescence emission intensity.

### 2.6. Detection of Early Neurological Reflexes

Twenty-four hours (24 h) after establishing the HIBI model, early neurological reflexes [24] including righting, cliff aversion, and geotaxis reflexes were evaluated in neonatal rats as follows. (1) The righting reflex was assessed by placing each neonatal rat in a supine position and recording the time (in seconds) to roll over to a normal prone position. For each neonatal rat, the test was performed three times to obtain a mean value. (2) The cliff aversion reflex was assessed by dangling the upper limbs of each neonatal rat from a board edge and recording the time to turn 90° away from the cliff edge. The maximum duration of observation was 20 s. Neonatal rats that did not turn 90° within the observation period were recorded at a time of 20 s. (3) The geotaxis reflex was measured by placing the head of each neonatal rat downward on a 40° inclined plate and recording the time required for turning around (with the head facing upward and >90° rotation). The maximum duration of observation was 20 s. Neonatal rats that did not turn within the observation period were recorded at a time of 20 s.

### 2.7. Morris Water Maze Test

The Morris water maze was used to study the effects on spatial learning and memory in rats [25, 26]. The test was performed at week 10 after establishing the HIBI model (postnatal days 70–74, representing rats in adulthood [27]). Rats were tested in a pool of 160 cm in diameter. The pool was filled with water at a depth of 40 cm. The rescue landing platform (8 cm in diameter) was submerged by 1 cm under the water surface in one of four quadrants. Water temperature was maintained at 23°C–24°C. Testing consisted of four consecutive training days during which four attempts (starts from four quadrants) were given to each rat to find the platform within 60 s. Each rat was given 20 min to rest between swimming trials. The training session occurred at the same time of day throughout the training and testing periods. A rat was placed in the water in the quadrant opposite to the landing, and the latency time (in seconds) spent by each animal to find the platform was recorded. If the rat was not able to locate and climb on the landing platform within the allotted time, the experimenter gently placed the animal on the platform and let the animal become accustomed to it for 20 s. On day 5 of probe training testing, the rescue platform was removed and the time spent in the quadrant where the landing used to be was recorded (allotted time 60 s). Only one attempt was given to each rat during the probe trial on day 5 of testing.

### 2.8. Western Blot Analysis

Rats were sacrificed by decapitation after the last H_2_ treatment, and the brain was microdissected and stored at -80°C. NGF-differentiated PC12 cells were washed with PBS and lysed in RIPA buffer (Proteintech, Rosemont, PA, USA) for 30 min on ice. The protein concentration in the extracts was determined using the BCA assay (Wellbio, Changsha, China). Equal amounts of protein (30 *μ*g) were separated by 10% sodium dodecyl sulfate polyacrylamide gel electrophoresis and transferred to polyvinylidene fluoride membranes (Millipore, Billerica, MA). Membranes were blocked in 5% skim milk powder dissolved in phosphate-bufferred saline with 0.5% Tween 20 in PBS (PBST) at 4°C overnight and then incubated with the following antibodies: anti-GFAP (1 : 1000; Abcam), anti-HMGB1 (1 : 1000; Abcam), anti-TLR4 (1 : 500; Proteintech, Rosemont, IL, USA), anti-phosphorylated nuclear factor kappa B (p-NF-*κ*B) p65 (1 : 1000; Abcam), anti-caspase3 (1 : 1000; Abcam), anti-BAX (1 : 1000; Abcam), anti-Bcl-2 (1 : 500; Proteintech), anti-p-p38 MAPK (1 : 1000; Abcam), anti-p-ERK1/2 (1 : 1000; Abcam), anti-p-JNK antibody (1 : 1000; Abcam), anti-Nrf2 (1 : 1000, Abcam), anti-HO-1 (1 : 1000; Abcam), anti-PGC-1*α* (1 : 1000; Abcam), and anti-SIRT1 (1 : 1000; Abcam). *β*-Actin (Proteintech) was used as the gel loading control. Following overnight incubation, blots were washed three times with PBST and then incubated with the secondary antibody (IRDye 800CW Goat Anti-Rabbit IgG, 1 : 10000; Invitrogen, Carlsbad, CA, USA). Proteins were detected using the Odyssey Infrared Imaging System (LI-COR Biosciences, Lincoln, NE, USA). Band intensity was quantified using ImageJ software.

### 2.9. Cell Culture and Differentiation

The PC12 cell line was obtained from American Type Culture Collection (Manassas, VA, USA) and maintained at 37°C in a humidified atmosphere containing 5% CO_2_ in high-glucose Dulbecco's Modified Eagle's Medium supplemented with 10% heat-inactivated fetal calf serum, 10% heat-inactivated horse serum, 100 kU/L penicillin, and 100 mg/L streptomycin. For differentiation, PC12 cells were plated at a low density on collagen-coated plastic in DMEM plus 1% horse serum and NGF (50 ng/mL) for 7 days.

### 2.10. PC12 Model of OGD

The cell culture was incubated at 37°C in a low oxygen incubator (1% O_2_, 94% N_2_, and 5% CO_2_) for 6 h (OGD). Then, glucose-free DMEM was replaced with normal medium, followed by incubation for 3/6/24 h (OGD/R) in standard culture gas (N_2_ as base gas, O_2_ 21%, and CO_2_ 5%). For the OGD/R+H2 group, cells were incubated for 3/6/24 h in standard culture gas with H_2_ gas (N_2_ as base gas, O_2_ 21%, CO_2_ 5%, and H_2_ 2–3%). Immediately after inducing OGD/R or OGD/R+H2, cells were collected for western blotting, quantitative PCR (qPCR), fluorescence-activated cell sorting (FACS), and immunofluorescence staining.

### 2.11. RNA Extraction and qPCR Analysis

All RNA was extracted from cells using a DNA/RNA Mini Kit (QIAGEN, Germantown, MD USA). Reverse transcription was done using the High Capacity cDNA Reverse Transcription Kit (Thermo Fisher Scientific, Waltham, MA USA). Quantitative measurements of target gene expression relative to 18 s RNA were performed in triplicate using the TaqMan Real-Time PCR assay following the manufacturer's recommendations in a Real-Time PCR system.

### 2.12. Annexin V and Propidium Iodide Staining Assay

Cells were detached and collected into a 15 mL centrifuge tube, washed twice with ice-cold PBS, and centrifuged at 1000 rpm for 5 min to remove the supernatant. A volume of 0.1 mL binding buffer containing 0.14 M NaCl, 2.5 mM CaCl_2_, and 0.01 M HEPES/NaOH pH 7.4 was added to resuspend the cells, followed by adding 5 *μ*L Annexin V-FITC and 5 *μ*L of 50 *μ*g/mL propidium iodide (PI) staining reagents. After mixing homogeneously and reacting at 25°C for 15 min in the dark, the apoptotic cell population was analyzed by a flow cytometer.

### 2.13. Statistical Analysis

Mean ± standard deviation was calculated for all parameters determined in this study. Repeated measures of two-way analysis of variance (ANOVA) were used for each parameter of the water maze task, followed by multiple *t*-test analyses. One-way ANOVA followed by Tukey's multiple-comparison and western blotting was used for histopathological parameters (volume and cell density). *P* < 0.05 was considered statistically significant.

## 3. Results

### 3.1. H_2_ Significantly Reduces Areas of Cerebral Infarction, Neuronal Loss, and Glial Activation in HIBI Rats

**Figure 2:**
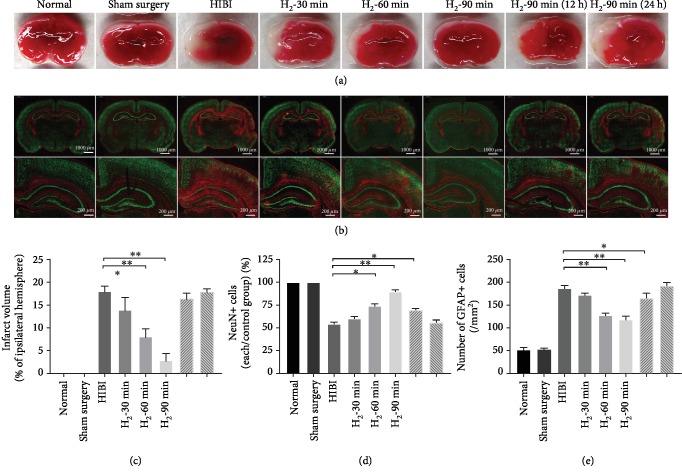
H_2_ reduced the area of infarction, neuronal loss, and glial activation in HIBI rats. (a) Representative results of TTC staining of each group. (b) Double-immunofluorescence staining (NeuN, green, expressed by neurons; GFAP, red, expressed by activated astrocytes) of each group (scale bar: 1000 *μ*m and 200 *μ*m). (c) Percentage of cerebral infarct volume in the ipsilateral hemisphere of different groups. Percentage of cerebral infarct volume was calculated as follows: percentage of cerebral infarct volume = cerebral infarct volume/normal cerebral hemisphere volume × 100%. Compared with the HIBI group, the percentage of cerebral infarct volume was significantly reduced in the H_2_ intervention groups (*n* = 8/group; ^∗^*p* < 0.05; ^∗∗^*p* < 0.01). (d, e) Bar graphs comparing relative NeuN immunoreactivity (NeuN+ cells) of each group/sham surgery group and the number of GFAP-immunoreactive cells (/mm^2^), respectively, in different groups (*n* = 8/group; ^∗^*p* < 0.05; ^∗∗^*p* < 0.01).

As shown in Figures 2(a) and 2(c), TTC staining showed no obvious white infarcts in either the left or right cerebral hemisphere in the normal and sham surgery groups but showed obvious white infarcts in the left cerebral hemisphere of the HIBI group, with ligation of the left common carotid artery. The area of cerebral infarction was significantly reduced with an increase in duration of H_2_ inhalation (^∗^*p* < 0.05; ^∗∗^*p* < 0.01). As shown in Figure 2(b), antibodies for two different biomarkers, neuronal nuclei (NeuN) and GFAP, were used in immunofluorescence staining to observe changes in neurons and astrocytes in the hippocampus. Compared with the normal and sham surgery groups, the immunoreactivity of NeuN was significantly reduced in the cortex and hippocampal CA3 regions of the HIBI group (^∗^*p* < 0.05; ^∗∗^*p* < 0.01; Figure 2(d)), which suggested that HIBI caused neuronal loss. In addition, the immunoreactivity of GFAP was significantly increased in the HIBI group (^∗^*p* < 0.05; ^∗∗^*p* < 0.01; Figure 2(e)), which suggested that astrocytes were activated. However, compared with the HIBI group, the immunoreactivity of NeuN was significantly increased in the cortex and hippocampal CA3 regions of the H_2_ inhalation groups, especially in the H_2_-90 min group, and the immunoreactivity of GFAP was significantly decreased in the H_2_ inhalation groups. Thus, H_2_ inhalation might protect neurons from injury and reduce activation of astrocytes. We also found that the decrease of neuronal loss was significantly associated with the duration and initiation time of H_2_ inhalation. H_2_ inhalation for 90 min immediately after HIBI had the most significant protective effect (^∗^*p* < 0.05; ^∗∗^*p* < 0.01), while 90 min H_2_ inhalation 24 h after HIBI had no significant protective effect on neuronal injury but did reduce activation of astrocytes, which suggests that astrocytes may be involved in the processes of HIBI.

### 3.2. H_2_ Improves Early Behavioral Reflexes in HIBI Rats

**Figure 3:**
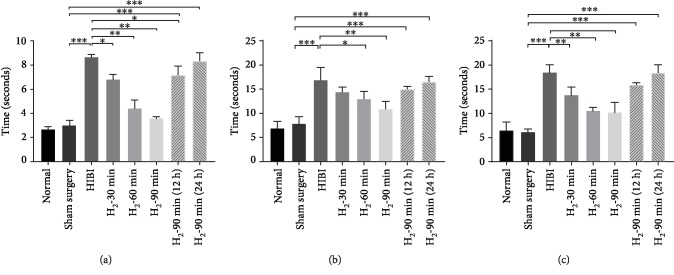
H_2_ improved the righting, geotaxis, and cliff aversion reflexes in HIBI rats. (a) Righting, (b) geotaxis, and (c) cliff aversion reflex performance in neonatal 8-day-old rats (*n* = 8/group; ^∗^*p* < 0.05; ^∗∗^*p* < 0.01).

To evaluate the neuroprotective effects of H_2_ on HI brain injury, we evaluated the righting, geotaxis, and cliff aversion reflexes of rats 24 h after HIBI. Neonatal rats in the HIBI group had a significantly slower righting reflex, and H_2_ inhalation improved the righting reflex in this group. Times for the righting reflex in the neonatal HIBI rats were significantly shortened with earlier initiation and extended duration of H_2_ inhalation (^∗^*p* < 0.05, ^∗∗^*p* < 0.01 vs. the sham surgery group ^∗∗∗^*p* < 0.01; Figure 3(a)). In assessment of the geotaxis reflex, neonatal rats in the HIBI group took significantly longer to turn from facing downward on the inclined plate, with the time to turn being three times greater than that of the sham surgery group. H_2_ intervention improved the geotaxis reflex (^∗^*p* < 0.05, ^∗∗^*p* < 0.01 vs. the sham surgery group ^∗∗∗^*p* < 0.01; Figure 3(b)). However, no significant difference was found between the H_2_-60 min and H_2_-90 min groups (*p* > 0.05). Improvement in time for turning was less pronounced with later H_2_ intervention in neonatal HIBI rats (Figure 3(b)). Assessment of the cliff aversion reflex showed results similar to those of the righting and geotaxis reflexes. Neonatal rats in the HIBI group had longer cliff avoidance times, and H_2_ inhalation decreased cliff avoidance time in this group. However, significant improvement in cliff avoidance time was only found in the H_2_-60 min and H_2_-90 min groups (^∗∗^*p* < 0.01 vs. the sham surgery group ^∗∗∗^*p* < 0.01; Figure 3(c)).

### 3.3. H_2_ Improved Learning and Memory of HIBI Rats during Adulthood

**Figure 4:**
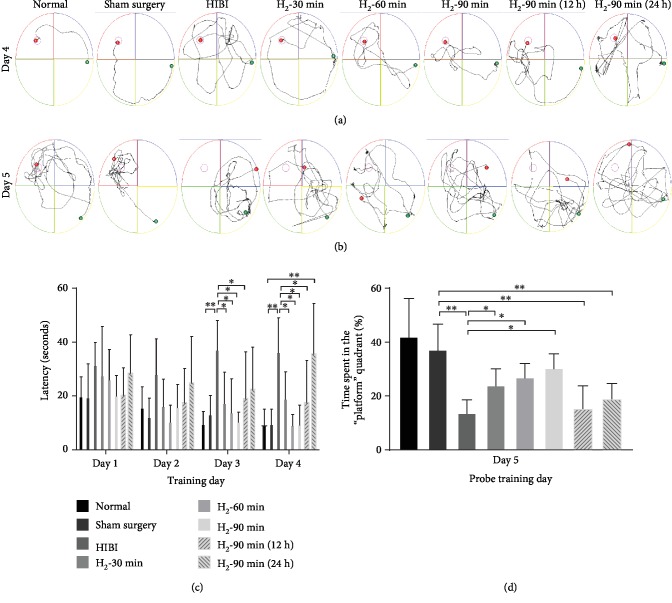
H_2_ improved learning and memory of HIBI rats during adulthood. (a) Diagrams indicating movement of rats in the normal (A), sham surgery (B), HIBI (C), H_2_-30 min (D), H_2_-60 min (E), H_2_-90 min (F), H_2_-90 min (12 h) (G), and H_2_-90 min (24 h) (H) groups to locate the escape platform during the day 4 training trial. (b) Diagrams indicating 60 s movement of rats in the normal (A), sham surgery (B), HIBI (C), H_2_-30 min (D), H_2_-60 min (E), H_2_-90 min (F), H_2_-90 min (12 h) (G), and H_2_-90 min (24 h) (H) groups to locate the escape platform during the day 5 probe trial. (c) Bar graph of latency to locate the escape platform during training trials on days 1 through 4 in different groups of rats (*n* = 8/group; ^∗^*p* < 0.05; ^∗∗^*p* < 0.01). (d) Spatial preference (percentage of time spent) in the escape platform quadrant during the day 5 probe trial (*n* = 8/group; ^∗^*p* < 0.05; ^∗∗^*p* < 0.01).

The spatial memory performance was evaluated using the Morris water maze. Rats were subjected to repeated Morris water maze tests 10 weeks after HI exposure or sham operation. The results showed that neonatal HIBI induced significant long-lasting cognitive deficits throughout brain maturation and adulthood. As shown in Figures 4(a) and 4(b), the mean latency to find the platform declined progressively during the period of training days in all animals except the HIBI and H_2_-90 min (24 h) groups. The latent period of the HIBI group was significantly longer than that of the control and sham surgery groups. H_2_ inhalation significantly shortened the latent period. This effect was correlated with the duration of H_2_ inhalation, and the H_2_-90 min group had the most significant improvement among the H_2_ intervention groups. Delays in initiation of H_2_ intervention reduced the neuroprotective effects of H_2_ against brain injury. The navigational memory of the rats was assessed by removing the escape platform on the fifth day of the Morris water maze test. Neonatal HIBI rats with H_2_ intervention in different groups demonstrated better navigational memory than the HIBI group, and the H_2_-60 min, H_2_-90 min, and H_2_-90 min (12 h) groups showed significant differences (^∗^*p* < 0.05; ^∗∗^*p* < 0.01; Figures 4(c) and 4(d)).

### 3.4. H_2_ Inhibits Activation of HMGB1 and TLR-4 Pathways in HIBI Rats

**Figure 5:**
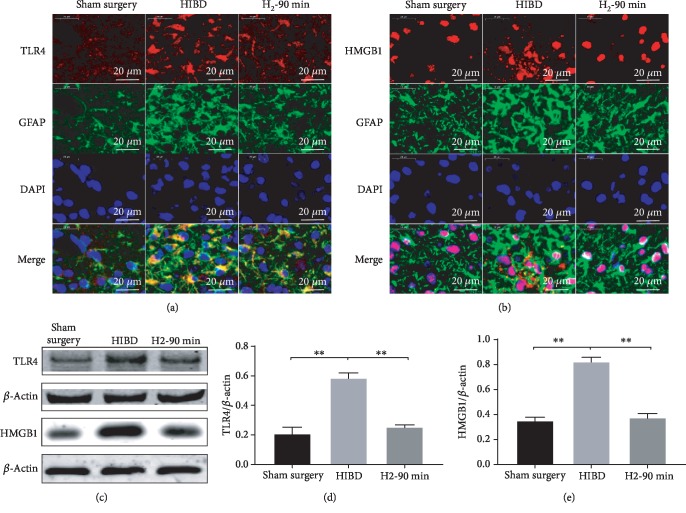
H_2_ inhibited HMGB1/TLR-4 expression in the hippocampal CA3 region of neonatal HIBI rats. (a) The representative images of TLR-4 (red) and glial fibrillary acidic protein (GFAP, green) and 4′,6-diamidino-2-phenylindole (DAPI, blue) immunofluorescence staining as well as merged immunofluorescent signals of all markers in the hippocampal CA3 region of sham surgery, HIBI, and H_2_-90 min groups (scar bar: 50 *μ*m). (b) Representative images of HMGB1 (red) and glial fibrillary acidic protein (GFAP, green) and 4′,6-diamidino-2-phenylindole (DAPI, blue) immunofluorescence staining as well as merged immunofluorescent signals of all markers in the hippocampal CA3 region of sham surgery, HIBI, and H_2_-90 min groups (scale bar: 50 *μ*m). (c) Western blot analysis of TLR-4, HMGB1, and *β*-actin proteins in the hippocampus of each group. (d, e) Bar graphs of the relative expression of TLR-4, HMGB1 in the hippocampus of each group (*n* = 3/group; ^∗∗^*p* < 0.01).

To explore the protective mechanism of H_2_ on HIBI, we assessed the expression of HMGB1, TLR-4, and NF-*κ*B-p65 in the hippocampus of neonatal rats. As shown in Figure 5, we found significantly enhanced expression of TLR-4 and GFAP in the HIBI group, suggesting that astrocytes were associated with enhanced TLR-4 signaling. In the HIBI+H_2_ groups, TLR-4 and GFAP expression was lower compared with the HIBI group and the longer the H_2_ inhalation, the lower the TLR-4 and GFAP expression. We also found that the HMGB1 expression was mainly localized in the nuclei. In the HIBI group, HMGB1 expression was significantly enhanced and detected outside the nuclei and GFAP expression was also enhanced. In the HIBI+H_2_ groups, GFAP expression was reduced compared with the HIBI group and HMGB1 expression was confined to the nuclei, suggesting that the secretion of HMGB1 was reduced after H_2_ inhalation (^∗∗^*p* < 0.01; Figure 5).

### 3.5. H_2_ Improves Hippocampal Bcl-2/BAX/Caspase-3 Protein Expression in HIBI Rats

**Figure 6:**
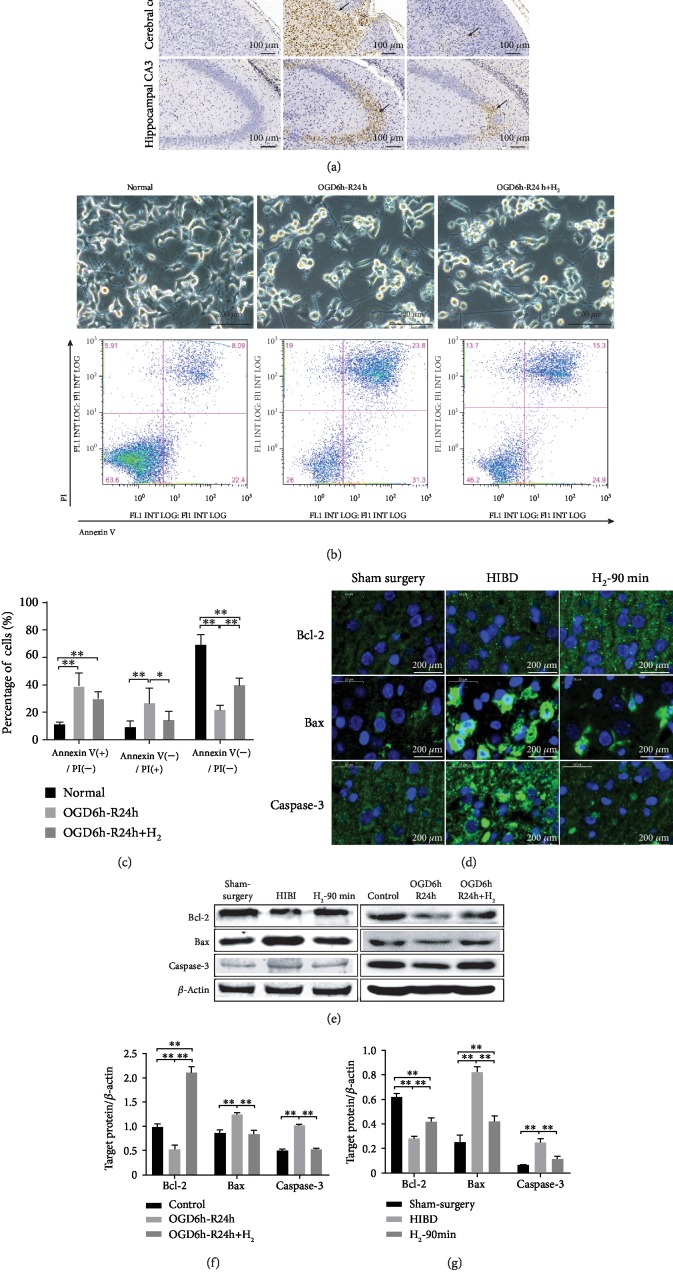
H_2_ inhibited apoptosis in HIBI rat brain neurons and NGF-differentiated PC12 cells. (a) Apoptotic cells were detected by TUNEL staining (dark brown, indicated by arrows) in the brains of neonatal rats 3 days after HIBI. (b) Images of NGF-differentiated PC12 cells were taken under an inverted microscope immediately after OGD/R. Then, apoptosis analysis was done by Annexin-V/PI double staining. (c) Data of viable (Annexin V-negative/PI-negative), early apoptotic (Annexin V-positive/PI-negative), and late apoptotic (Annexin V-positive/PI-positive) cells from three independent experiments (^∗^*p* < 0.05; ^∗∗^*p* < 0.01). (d) Representative immunofluorescent signals (green) of Bcl-2, BAX, and caspase-3 with DAPI nuclear counterstain (blue) (scale bar: 20 *μ*m). (e) Western blot analysis of *β*-actin, Bcl-2, BAX, and caspase-3 proteins in each group. (f, g) Bar graph showing the relative expression of Bcl-2, BAX, and caspase-3 in each group (*n* = 3/group; ^∗∗^*p* < 0.01).

As shown in Figure 6(a), TUNEL staining showed no detectable neuronal apoptosis in the sham surgery group but showed elevated apoptosis neurons in the cortex and hippocampal CA3 region of the HIBI group. The number of apoptotic neurons was significantly reduced in the H_2_-90 min group. The Annexin-V/PI double staining assay further confirmed that H_2_ attenuated apoptotic events in NGF-differentiated PC12 cells following OGD/R. Representative assay results are shown in Figure 6(b), and statistical analyses are summarized in Figure 6(c). Both early (Annexin V^+^/PI^−^) and late (Annexin V^+^/PI^+^) stages of apoptotic PC12 cells increased in the OGD/R group, while H_2_ reduced the number of apoptotic PC12 cells (^∗^*p* < 0.05; ^∗∗^*p* < 0.01). Bcl-2 and BAX genes have been recognized as the two most important regulatory genes that are functionally antagonistic to each other in the regulatory processes of apoptosis. Bcl-2 inhibits apoptosis, while BAX promotes apoptosis. Caspase-3 is the most critical apoptosis executioner protease. In this study, the HIBI group showed downregulated Bcl-2 protein expression and upregulated BAX and caspase-3 protein expression (Figure 6(d)). Bcl-2 protein expression in the H_2_ intervention groups was higher than that in the HIBI and OGD/R groups, while BAX and caspase-3 protein expression in the H_2_ intervention groups was lower than that in the HIBI and OGD/R groups (^∗∗^*p* < 0.01; Figures 6(e)–6(g)).

### 3.6. H_2_ Regulates the Expression of MAPK/Nrf2/HO-1

**Figure 7:**
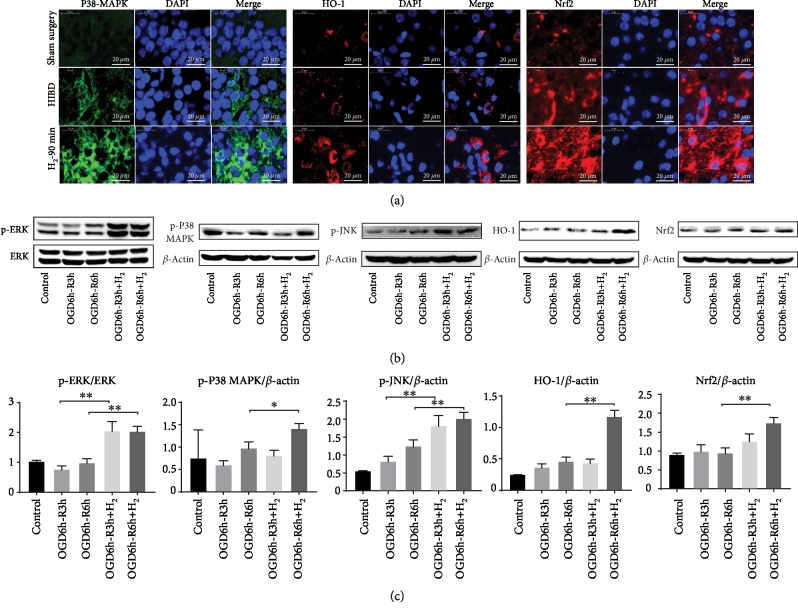
Effects of H_2_ gas on MAPK/Nrf2/HO-1 protein expression. (a) Representative images of p-p38 MAPK (green), Nrf2 (red), HO-1 (red), and DAPI (blue) immunofluorescence staining of the rat cortex in the sham surgery, HIBI, and H_2_-90 min groups (scar bar: 20 *μ*m). (b) p-ERK, p-P38 MAPK, p-JNK, Nrf2, and HO-1 protein expressions were analyzed by western blot analysis in each group of *β*-NGF-differentiated PC12 cells. (c) Bar graph showing the relative expression of p-ERK, p-p38 MAPK, p-JNK, Nrf2, and HO-1 in each group (*n* = 3/group; ^∗^*p* < 0.05; ^∗∗^*p* < 0.01).

We examined the expression of p-p38 MAPK, HO-1, and Nrf2 in the cortex of HIBI rats. As shown in Figure 7(a), H_2_ significantly enhanced the p-p38 MAPK, HO-1, and Nrf2 expression in the HIBD rat cortex. As shown in Figures 7(b) and 7(c), H_2_ significantly enhanced the p-p38 MAPK, p-ERK1/2, p-JNK, HO-1, and Nrf2 expression in NGF-differentiated PC12 cells following OGD/R (^∗∗^*p* < 0.01).

### 3.7. Role of p38 MAPK, ERK, and JNK in HO-1 Expression Induced by H_2_

**Figure 8:**
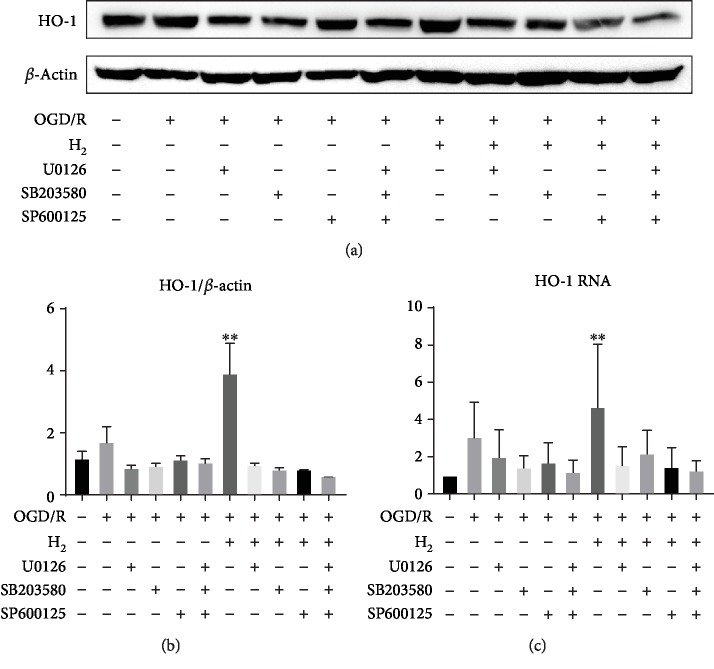
Involvement of MAPK in the expression of HO-1 induced by H_2_ gas in differentiated PC12 cells. *β*-NGF-differentiated PC12 cells were pretreated with U0126 (10 mM), SB203580 (20 mM), or SP600125 (10 mM) for 1 h followed by oxygen-glucose deprivation (6 h) and then reperfusion with or without H_2_ gas. The expression of HO-1 protein and mRNA was measured by western blotting and qPCR, respectively. (a) Western blot analysis of *β*-actin and HO-1 proteins in each group. (b) Protein expression of HO-1 is shown as the HO-1/*β*-actin ratio for each sample. (c) The mRNA expression of HO-1 was normalized with 18S to determine the relative expression ratio (log2 fold) for each group (*n* = 3/group; ^∗∗^*p* < 0.01).

The effects of p38 MAPK, ERK, and JNK inhibition on the expression of HO-1 are shown in Figure 8. The p38 MAPK pathway inhibitor SB203580, ERK inhibitor U0126, and JNK inhibitor SP600125 significantly reduced OGD/R-induced injury, and H_2_ plus OGD/R injury induced HO-1 expression (^∗∗^*p* < 0.01).

### 3.8. Role of HO-1 in PCG1-*α* and SIRT1 Expression Induced by H_2_

**Figure 9:**
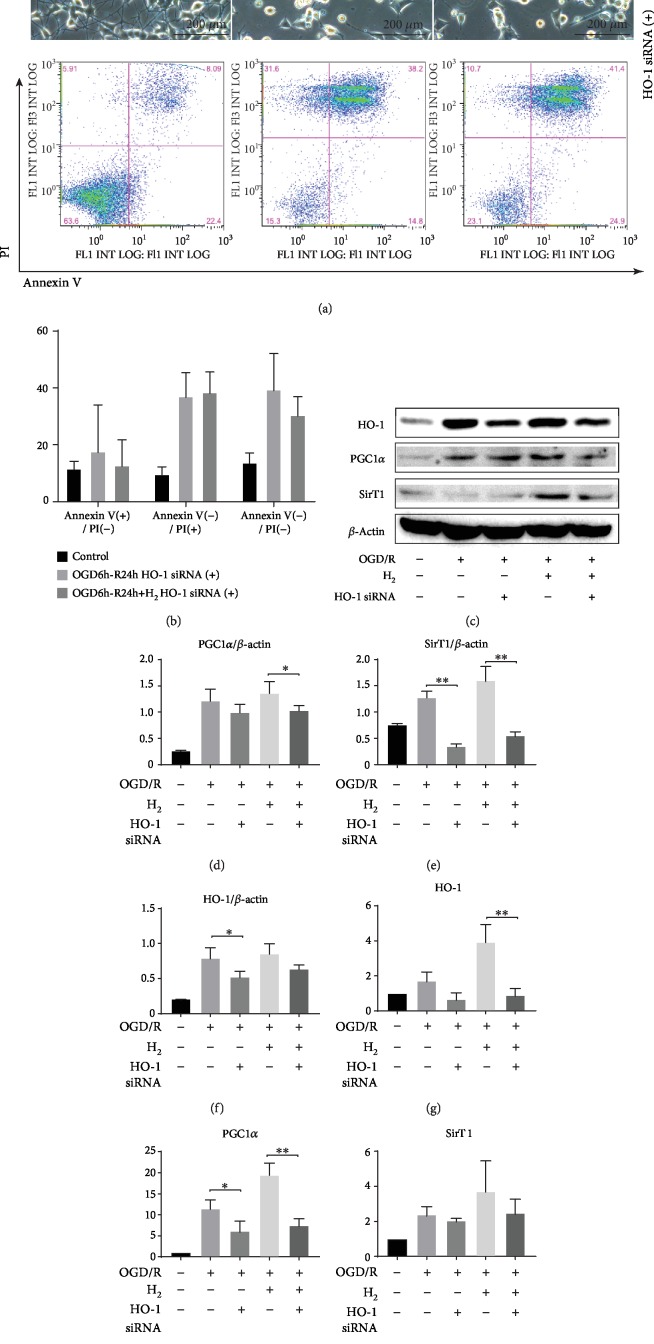
Knockdown of HO-1 expression in the HO-differentiated PC12 cells was followed by OGD and reperfusion with or without H_2_ gas. *β*-NGF-differentiated PC12 cells were pretreated with HO-1 siRNA for 24 h followed by OGD (6 h) and then reperfusion with or without H_2_ gas. (a) Percentage of apoptotic cells (provided for each condition) was determined by combined Annexin V/PI staining and FACS analysis. (b) Data of early apoptotic (Annexin V-positive/PI-negative), late apoptotic (Annexin V-positive/PI- positive), and dead (Annexin V-negative/PI-negative) cells are summarized for three independent experiments. (c) Western blot analysis of *β*-actin, HO-1, PGC-1*α*, and SIRT1 proteins in each group. (d–f) The protein expression of HO-1, PGC-1*α*, and SIRT1 is shown as the ratio of the protein to *β*-actin for each sample. (g–i) The mRNA expression of each gene was normalized with 18S to determine the relative expression ratio (log2 fold) for each group (*n* = 3/group; ^∗^*p* < 0.05; ^∗∗^*p* < 0.01).

The effects of HO-1 silencing on apoptosis and expression of PGC-1*α* and SIRT1 are shown in Figure 9. NGF-differentiated PC12 cells treated with HO-1 small interfering RNA (siRNA) showed increased apoptosis events following OGD/R injury, and H_2_ did not attenuate apoptosis (Figures 9(a) and 9(b)). As shown in Figures 9(c)–9(i), HO-1 siRNA markedly decreased the expression of HO-1, PGC-1*α*, and SIRT1 induced by H_2_ in NGF-differentiated PC12 cells following OGD/R (^∗^*p* < 0.05; ^∗∗^*p* < 0.01).

## 4. Discussion

Neonatal HIE is the most common cause of neonatal death and disability, often causing motor, sensory, and cognitive impairments [28]. Mild hypothermia has been recognized as an effective treatment to reduce HIE mortality. However, the disability and mortality rates of HIE are still very high [29]. Therefore, studying new effective and safe treatments for HIE is an important task in medical research. The neuroprotective effects of H_2_ treatment against HIBI are controversial. Cai et al. [9] found that H_2_ therapy significantly reduced the number of TUNEL-positive cells and attenuated caspase activity, suggesting that H_2_ gas exerts neuroprotective effects by inhibiting cellular apoptosis after HI. However, in another study of HI, hydrogen treatment failed to show neuroprotective effects, possibly as a result of the severity of injury [10]. In both studies, HIBI rats received H_2_ inhalation only once, without systematically studying possible pathways. Therefore, we considered whether H_2_ gas treatment of HIE is related to the degree of HI and the duration of inhalation.

In this study, we reported for the first time that the protective effects of H_2_ inhalation are related to the start time and duration. First, anatomical, cerebral, and hippocampal infarction, neuronal loss, and astrocyte activation were found in neonatal rats during the early HIBI stage; in H_2_ inhalation groups, these injuries were alleviated, with the most significant results occurring in the H_2_-90 min group (Figure 2). We also conducted short-term behavioral analyses of rats and showed that HIBI decreased these neurological reflexes, which suggested impaired neuronal function. However, H_2_ alleviated this impairment in neonatal rats (Figure 3). The Morris water maze test, which was conducted once the neonatal rats reached adulthood (Figure 4), evaluated navigational abilities in learning and memory. The results indicated that rats in the HIBI group had significant impairments in learning and memory, while H_2_ intervention improved performance. Shortening the duration of H_2_ inhalation and delaying H_2_ intervention reduced the protective effects of H_2_. Encouraged by these protective effects, we further investigated the related mechanisms and found that the mechanisms underlying the protective effects included anti-inflammation, antiapoptosis, and antioxidative stress.

The main mechanisms of neonatal HIBI include oxidative stress, excitability, inflammation, and apoptosis [2]. Many researchers believe that similar to nitric oxide, hydrogen sulfide, and carbon monoxide, H_2_ may be an important bioactive gas [30, 31]. There are no reported side effects with H_2_, which may confer a unique advantage in disease prevention and treatment [31]. In addition, human safety has been demonstrated and standards have been established for inhalation of high H_2_ concentrations; high-pressure H_2_ gas is used in deep diving gas mixes to prevent decompression sickness and arterial gas thrombi [32].

Many neurological studies have associated the activation of astrocytes with CNS injury [33]. Activation of glial cells may induce subsequent inflammatory responses, attributed to neuronal injury and repair mechanisms later in life and resulting in neurodevelopmental disorder [34]. In this study, we found that HI activated astrocytes in the hippocampus of HIBD rats and H_2_ inhalation reduced the activation of astrocytes (Figure 5). These results suggest that H_2_ may inhibit subsequent inflammatory responses by reducing glial cell activation. We also found that H_2_ suppressed the expression of HMGB1 and TLR-4 (Figure 5). The HMGB1/TLR-4/NF-*κ*B signaling pathway is well known to be a central regulator of several types of inflammatory responses including astrocyte-mediated neuroinflammation [35].

Apoptosis plays a more prominent role in the progress of ischemic brain injury in neonatal rodents and humans than in adult brain ischemia [36]. HI can promote a series of pathological changes in neurons, which result in ectopic expression of the death promoter BAX, leading to the formation of the apoptosome. Then, the apoptosome activates procaspase-9, which is followed by the activation of procaspase-3 and ultimately cell death [37, 38]. As members of the Bcl-2 family of proteins, BAX and Bcl-2 serve as a class of apoptosis regulators at its early stages. Bcl-2 is an antiapoptotic protein that counteracts the proapoptotic effects of BAX. Above all, an appropriate ratio of BAX/Bcl-2 can maintain a homeostatic state in cells and ensure cell survival [39]. In our study, we found that H_2_ could significantly suppress the expression of BAX and caspase-3 and concurrently promote the expression of antiapoptotic protein Bcl-2 in neurons, which strongly supports the notion that H_2_ has an anticell death activity (Figure 6).

**Figure 10:**
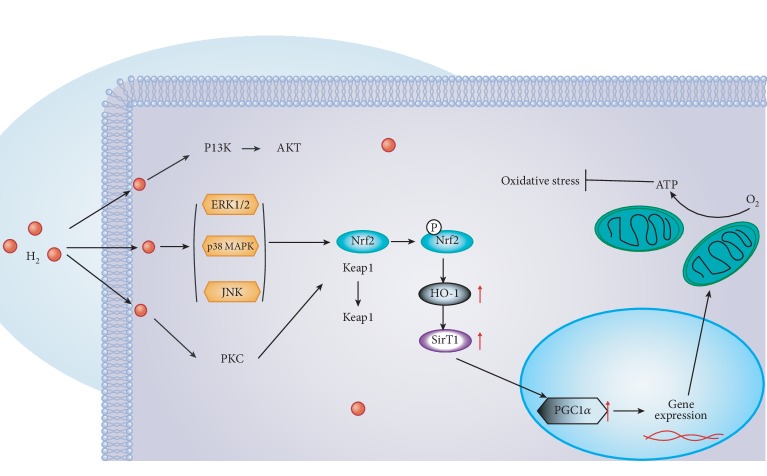
The proposed mechanism by which H_2_ gas regulate the MAPK/Nrf2/HO-1 pathway. Step 1: H_2_ gas upregulates the expression of MAPKs (ERK1/2, P38 MAPK, and JNK). Step 2: HO-1 can be expressed by stimuli mainly via MAPK-dependent Nrf2 activation. Step 3: HO-1 inhibits oxidative stress and increases SIRT1 expression. Step 4: SIRT1 directly deacetylates PGC-1*α*. In summary, the functional outcome is decreased apoptosis and oxidative stress.

Recent studies have proposed that oxidative injury to vital cellular structures contributes to the pathogenesis of HIE [40]. To explore the potential oxidative stress signaling pathways underlying the oxidative stress response after HIBI, we investigated the expression of antioxidant enzyme HO-1. Recent studies have demonstrated that HO-1 activation contributes to the inhibition of glial hyperactivation and protection against gliosis-induced neuronal damage in the brain [41, 42]. HO-1 activation not only confers antioxidative effects but also modulates redox homoeostasis and regulates neuroinflammatory conditions in activated glial cells [43]. PGC-1*α* has been demonstrated to be essential for the regulation of mitochondrial oxidative stress and biogenesis [44]. To explore the potential oxidative stress signaling pathways underlying the oxidative stress response after HIBI, we investigated MAPKs, HO-1, and Nrf2 in neurons (Figure 7) and suggested that the HO-1 system is one of the major protective pathways of H_2_ in HI-induced injury. Another novel finding of this study is the importance of MAPK/HO-1/PGC-1*α* signaling in H_2_'s neuroprotective actions. H_2_ activated MAPKs (ERK1/2, p38 MAPK, and JNK) and increased HO-1 expression, and MAPK inhibitors blocked the increase of HO-1 induced by H_2_ (Figure 8). HO-1 siRNA inhibited HO-1/PCG1-/SIRT1 expression and enhanced PC12 cell apoptosis induced by OGD/R (Figure 9). We believe that H_2_ exerts cytoprotection against HI by reducing neuron apoptosis and regulating the MAPK/HO-1/PCG1-*α* pathway (Figure 10).

## 5. Conclusions

Our results demonstrate that H_2_ inhalation is a potential treatment for neonatal HIE. We propose early (<12 h after HIE or HIBI) and prolonged (>60 min) H_2_ inhalation to protect against HIBI. Furthermore, our study suggests possible underlying mechanisms mediated by the suppression of HMGB1, TLR-4, BAX, and saspase-3 and activation of the MAPK/HO-1/PGC-1*α* pathway. We believe that the protective effects of H_2_ may be related to anti-inflammation, antiapoptosis, and antioxidative stress.

## Data Availability

The datasets used and/or analyzed during the current study are available from the corresponding authors on reasonable request.
