# Corrigendum to “Hydrogen Gas Attenuates Hypoxic-Ischemic Brain Injury via Regulation of the MAPK/HO-1/PGC-1a Pathway in Neonatal Rats”

**DOI:** 10.1155/2021/3539415

**Published:** 2021-01-23

**Authors:** Peipei Wang, Mingyi Zhao, Zhiheng Chen, Guojiao Wu, Masayuki Fujino, Chen Zhang, Wenjuan Zhou, Mengwen Zhao, Shin-ichi Hirano, Xiao-Kang Li, Lingling Zhao

**Affiliations:** ^1^Department of Pediatrics, The Third Xiangya Hospital, Central South University, Changsha, China; ^2^Division of Transplantation Immunology, National Research Institute for Child Health and Development, Tokyo, Japan; ^3^AIDS Research Center, National Institute of Infectious Diseases, Tokyo, Japan; ^4^MiZ Co., Ltd., Kanagawa, Japan

In the article titled “Hydrogen Gas Attenuates Hypoxic-Ischemic Brain Injury via Regulation of the MAPK/HO-1/PGC-1a Pathway in Neonatal Rats” [1], the authors identified that there was an error in the western blot images of Figure 5(c) where the incorrect images were presented for the second *β*-actin bands. The authors confirm that this does not affect the conclusions of the article, and the corrected Figure 5 is as follows:

## Figures and Tables

**Figure 1:**
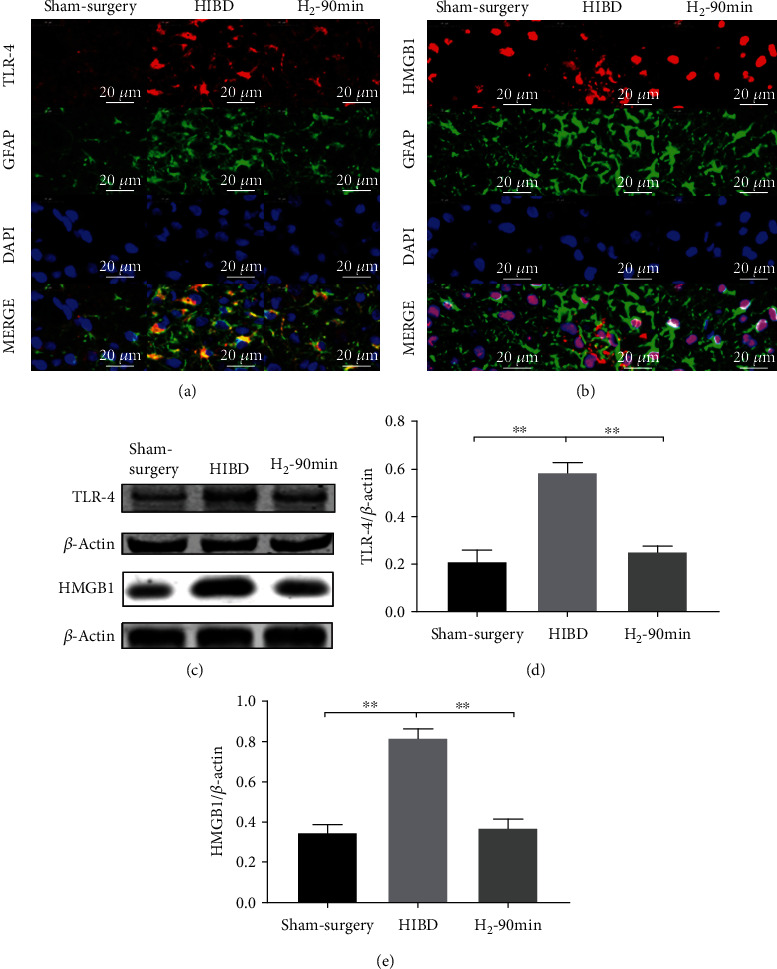
H_2_ inhibited HMGB1/TLR-4 expression in the hippocampal CA3 region of neonatal HIBI rats. (a) The representative images of TLR-4 (red) and glial fibrillary acidic protein (GFAP, green) and 4,6-diamidino-2-phenylindole (DAPI, blue) immunofluorescence staining as well as merged immunofluorescent signals of all markers in the hippocampal CA3 region of the sham surgery, HIBI, and H_2_-90 min groups (scale bar: 50 *μ*m). (b) Representative images of HMGB1 (red) and glial fibrillary acidic protein (GFAP, green) and 4,6-diamidino-2-phenylindole (DAPI, blue) immunofluorescence staining as well as merged immunofluorescent signals of all markers in the hippocampal CA3 region of the sham surgery, HIBI, and H_2_-90 min groups (scale bar: 50 *μ*m). (c) Western blot analysis of TLR-4, HMGB1, and *β*-actin proteins in the hippocampus of each group. (d, e) Bar graphs of the relative expression of TLR-4, HMGB1 in the hippocampus of each group (*n* = 3/group; ^∗∗^*p* < 0.01).
